# Peer-Selected “Best Papers”—Are They Really That “Good”?

**DOI:** 10.1371/journal.pone.0118446

**Published:** 2015-03-19

**Authors:** Jacques Wainer, Michael Eckmann, Anderson Rocha

**Affiliations:** 1 Computing Institute, University of Campinas, Campinas, São Paulo, Brazil; 2 Department of Mathematics and Computer Science, Skidmore College, Saratoga Springs, NY, United States of America; Katholieke Universiteit Leuven, BELGIUM

## Abstract

**Background:**

Peer evaluation is the cornerstone of science evaluation. In this paper, we analyze whether or not a form of peer evaluation, the pre-publication selection of the best papers in Computer Science (CS) conferences, is better than random, when considering future citations received by the papers.

**Methods:**

Considering 12 conferences (for several years), we collected the citation counts from Scopus for both the best papers and the non-best papers. For a different set of 17 conferences, we collected the data from Google Scholar. For each data set, we computed the proportion of cases whereby the best paper has more citations. We also compare this proportion for years before 2010 and after to evaluate if there is a propaganda effect. Finally, we count the proportion of best papers that are in the top 10% and 20% most cited for each conference instance.

**Results:**

The probability that a best paper will receive more citations than a non best paper is 0.72 (95% CI = 0.66, 0.77) for the Scopus data, and 0.78 (95% CI = 0.74, 0.81) for the Scholar data. There are no significant changes in the probabilities for different years. Also, 51% of the best papers are among the top 10% most cited papers in each conference/year, and 64% of them are among the top 20% most cited.

**Discussion:**

There is strong evidence that the selection of best papers in Computer Science conferences is better than a random selection, and that a significant number of the best papers are among the top cited papers in the conference.

## Introduction

Peer evaluation is the cornerstone of scientific evaluation focusing on two main aspects: the evaluation of scientists and the evaluation of their scientific work as reported in papers. Evaluation of scientists by peers is a central aspect of hiring, promotion, granting awards and distinctions, and it is an important aspect of most research grant evaluation. The peer evaluation, or peer review, of papers and manuscripts, in turn, is a much more frequent task—tens to hundreds of thousands manuscripts are evaluated by peers each year as the main part of the scientific publication process. A different form of peer evaluation of papers is the selection of particularly noteworthy papers. Some journals (for example in Civil Engineering and in Finance/Economics) award a yearly best paper selected from the papers published in the journal in the previous year. The Faculty of 1000 (F1000) (http://f1000.com/prime) selects and recommends noteworthy papers in the biomedical literature. In these examples of peer evaluation of noteworthy or “best” papers, the selection happens after the paper’s publication, and thus the peers may have some information about how the paper has been accepted by readers.

Finally, a slightly different form of peer evaluation of papers is to select, among accepted but yet unpublished papers, which ones are the “best”. This practice is somewhat common in some scientific disciplines, such as Computer Science, Engineering, and Management, which have a strong tradition of publication of scientific papers in conferences. Usually the conference selects one or more of the accepted papers as the “best” for that year. The selection is performed before the papers are published and presented, hence it is not based on the reception of the papers, but on the peers’ understanding and belief of their noteworthiness. This research will evaluate whether the papers selected by peer evaluation as “best” in Computer Science conferences are indeed “good”.

There are at least two measures for evaluating how important a paper is: how much it influences other people’s research, usually measured by citations received in the scientific literature, and how much it changes the practices of non-scientists. A paper may change how physicians treat their patients (in fact one of the F1000 classifications is that a paper is noteworthy because it “changes clinical practice”), or an Economics or Sociology paper may change government policies. In these cases, the practical importance of the paper may not be reflected in the number of citations received. In Computer Science, we are not aware of research that links publication of papers to changes in the practice of programmers or system designers, hence we will constrain our analysis to the “scientific importance” of a paper, and measure it by the number of citations received over time. We will evaluate how many more citations a “best” paper receives in comparison to the other papers of the same conference and year. In particular, we are interested in answering the following questions:

- Is the peer selection of best papers significantly different than random? In this case, a best paper should receive, on average, more citations than a random paper from the same conference and year.
- If the peer selection does indeed select papers that will receive, on average, more citations than a random paper, is it the case that the best paper is benefiting from a propaganda effect of being named a best paper?
- What proportion of the best papers is among the top cited for each conference and year?

### Related research

There has been a large volume of publications that discuss peer evaluation of scientists, the correlation or non-correlation of their evaluation and standard bibliometric measures [1–5]. Furthermore there is a large set of proposals of new bibliometric measures that aim at better evaluating scientists, including the h-index [6] and its many variations [7–9]. Finally, there is also a large volume of research and commentaries about manuscript peer evaluation. We will not extend on this issue for the sake of brevity.

Regarding one form of paper evaluation, the selection of noteworthy published papers, one must first note that there are two approaches to research in this area. One may consider that peer evaluation is the gold standard, and use it to evaluate how “correct” a standard measure such as citation count is. Or one may consider that citation count is the *a posteriori* correct measure of the importance of a paper, and use it to evaluate how correct the peer decision is, which was based on limited information of the impact of the paper on other readers. In this paper, we follow the latter approach.

Sen and Patel [10] study the citations received by the best papers awarded by the American Society of Civil Engineers (ASCE) from 1978 to 2002. The ASCE has six prizes, some of them general and others that are area specific. The awards are given each year to papers published in any of its 32 journals. The authors point out that 24.5% of the papers were uncited (using data from Web of Science), and use that as a criticism of using citation count as a measure of quality or importance of a paper (following the approach of peer evaluation as gold standard).

Coupé [11] analyzes the citation counts of papers awarded as best papers from seven finance and economics journals and concludes that:

- a best paper has a significantly higher number of citations than the median paper in the same volume;
- in 25% of the cases, the best paper was the highest cited paper in the volume;
- in a small majority of cases, the best paper has a higher citation count than the runner up.

The paper also studies the influence of other factors on receiving the award, such as length of the paper, number and gender of authors, and so on.

Faculty of 1000 is a service that recommends papers in life sciences based on the evaluation of 5,000 experts. Each expert may recommend any published paper, and classify it as “Good”, “Very Good” or “Exceptional” and must further classify the paper into groups, such as “changes clinical practice” or “good for teaching” and so on. A paper can receive recommendations from more than one expert, which adds to its ranking.

Wardle [12] compares the citations of 103 Ecology papers published in 2005, which were selected by F1000, 23 of them with F1000 score higher than 3, with 1,427 non-selected papers from the same journals in the same year. The selected papers had mean and median citation counts higher than the remaining papers (no statistical significance was provided), but the author claims “difference is not substantial, and the ability of F1000 to predict those papers that go on to have the greatest impact is poor.” [12]. In particular, none of the selected papers were among the top 11 most cited papers in the journals, and a large number of selected papers, even papers with scores larger than 2, were among the bottom 50% in the rank of cited papers.

Bornmann and Leydesdorff [13] correlate the F1000 rankings of 125 papers published in 2008 with seven bibliometric measures. The correlation to citations received by the paper is of particular interest for this research. The correlation discovered is low (*r* ^2^ = 0.20), which indicates that further positive evaluations by F1000 does not result in a higher number of citations. Notice that this research does not compare non-selected papers with selected papers by F1000; it only compares the correlation of “better ranking” to more citations.

Waltman and Costas [14] analyze almost all of the F1000 recommendations and also find a low correlation between recommendations (measured as the number of F1000 recommenders and highest recommendation level) and citations. On the other hand, around 50% of the recommended papers are among the top 10% cited papers.

Michayluk and Zurbruegg [15] study the difference in citation counts of lead papers in four finance journals, as compared to non-lead papers, under the assumption that the choice of a paper to lead the journal issue is an implicit evaluation by the editors of the quality or “interest” in the paper. The research discovered that for the 1998–2009 period, lead papers have consistently more citations than non-lead papers in the same journal. Different than the selection of best papers in a journal, the choice of lead article is made before publication, without any information on how the paper was received by the readers. The timing of this peer evaluation is similar to the choice of best papers at computer science conferences.

To our knowledge, there is no other published work that compares the impact of best papers to non-best papers at computer science conferences. The only work we found that shows the citation counts of best papers in computer science conferences and ranks their citation counts among other papers at that conference instance is http://arnetminer.org/conferencebestpapers. They rank the papers according to citation counts and show the top three per conference instance as well as the rank and citation count of the best papers.

We first became aware of that page in June 2014. We did our data collection and analysis independently and without knowledge of that webpage. It is worth noting, however, that our analysis is significantly different and one would likely draw different conclusions from that page vs. our statistical analysis. For example, from that page, one might draw the conclusion that best papers are generally not cited as well as non-best papers. See, for example, for AAAI 2012, the two best papers are ranked 59 & 174 in number of citations. However, our statistical analysis concludes that there is a high probability that the best paper will have more citations than a random paper in the same conference instance.

### Conferences in Computer Science

Computer Science (CS) research practices place much more value on scientific communication through conference and meetings than other scientific areas [16–19]. There are hundreds to thousands of CS conferences yearly, each with attendance from 50 to more than 15,000 (for instance, ACM Sigraph reported 21,000+ attendees in 2012. Conferences are mainly focused on specific sub-areas of CS. Almost all conferences publish proceedings that are available on the Internet; most conferences are sponsored and/or supported by international associations such as ACM, IEEE, or national associations. Submissions to CS conferences are always in the form of full papers ranging from 5 to 12 pages (as opposed to abstracts only in other areas) and acceptance rates in conferences range from 50% to 15% (as opposed to the “all submissions are accepted” policy common in some other areas).

In fact there is a perceived correlation between the prestige of a conference and lower acceptance rates, although one empirical evaluation shows that the correlation is not real [20]. Freyne et al. [20] showed that papers in prestigious CS conferences receive as many citations (as measured by Google Scholar) as papers in mid-ranking journals.

In 2008, Web of Science (WOS) added some selected conferences to its indexed sources. Bar-Ilan [17] showed that 40% of the citations to highly cited CS researchers came from papers published in conferences (the ones indexed by WOS). On the other hand, Wainer et al. [21] showed that, for faculty from very prestigious CS departments, on average, 47% of the researcher’s publications in conference proceedings is not included in the WOS (with proceedings).

## Methods and Materials

Jeff Huang at Brown University maintains a website at http://jeffhuang.com/best_paper_awards.html with the list of best papers for the CS conferences below. The list below contains the conference name and its abbreviation. We got our list of which papers were the best from that site.

- AAAI Conference on Artificial Intelligence (AAAI)
- Annual Meeting of the Association for Computational Linguistics (ACL)
- ACM SIGCHI Conference on Human Factors in Computing Systems (CHI)
- ACM International Conference on Information and Knowledge Management (CIKM)
- IEEE Conference on Computer Vision and Pattern Recognition (CVPR)
- Annual Symposium on Foundations of Computer Science (FOCS)
- ACM Foundations of Software Engineering Conference (FSE)
- International Conference on Computer Vision (ICCV)
- International Conference on Machine Learning (ICML)
- International Conference on Software Engineering (ICSE)
- International Joint Conference on Artificial Intelligence (IJCAI)
- International Conference on Computer Communications (INFOCOM)
- ACM SIGKDD Conference on Knowledge Discovery and Data Mining (KDD)
- ACM International Conference on Mobile Computing and Networking (MOBICOM)
- USENIX Symposium on Networked System Design and Implementation (NSDI)
- USENIX Symposium on Operating Systems Design and Implementation (OSDI)
- ACM Programming Language Design and Implementation (PLDI)
- ACM Symposium on Principles of Database Systems (PODS)
- IEEE Symposium on Security and Privacy (S&P)
- ACM Annual Conference of the Special Interest Group on Data Communication (SIGCOMM)
- ACM Annual Conference of the Special Interest Group on Information Retrieval (SIGIR)
- ACM Annual Conference of the Special Interest Group for the Computer Systems Performance Evaluation (SIGMETRICS)
- ACM Annual Conference of the Special Interest Group on Management of Data (SIGMOD)
- ACM-SIAM Symposium on Discrete Algorithms (SODA)
- ACM Symposium on Operating Systems Principles (SOSP)
- ACM Symposium on the Theory of Computing (STOC)
- ACM Symposium on User Interface Software and Technology (UIST)
- International Conference on Very large Data Bases (VLDB)
- International Conference on the World Wide Web (WWW)

The list includes very prestigious conferences in different areas of Computer Science. AAAI and IJCAI are in the general area of Artificial Intelligence; ACL is in Natural Language Processing; CHI and UIST are in User Interfaces; CIKM and SIGIR are in Information Retrieval; CVPR and ICCV are in Computer Vision; FOCS, STOC, and SODA are in Theory; FSE and ICSE are in Software Engineering; ICML and KDD are in Machine Learning; INFOCOM, MOBICOM, NSDI, and SIGCOMM are in Networking; OSDI and SOSP are in Operating Systems; PLDI is in Programming Languages; PODS, SIGMOD, and VLDB are in Databases; S&P is in Computer Security; SIGMETRICS is on System Performance, and WWW is in the World Wide Web.

In this paper, we will call AAAI, for example, a conference, and the AAAI that happened in 2002, which selected one best paper, a *conference instance*.

### Scopus data

Scopus explicitly indexes the following conferences from the list above: AAAI, CIKM, FSE, ICCV, ICSE, INFOCOM, KDD, MOBICOM, PODS, SIGMOD, SODA, and STOC. We collected the Scopus citation counts received for each of the best and non-best papers in these conferences for the years 1998 to 2012 inclusive. The Scopus data were collected in March 2013.

The Scopus data refer to 19,421 papers, of which 168 are best papers. Table 1 shows the distribution of the number of best and non-best papers for each conference instance.

**Table 1:** Specifics of the Scopus data. The first figure in each cell is the number of non-best papers in the conference instance. The figure in parenthesis is the number of best papers.

	AAAI	CIKM	FSE	ICCV	ICSE	INFOCOM	KDD	MOBICOM	PODS	SIGMOD	SODA	STOC
1998				165(2)		171(1)			30(2)			
1999				175(2)		182(1)			33(1)			
2000						191(1)			27(1)			
2001				217(2)		191(1)			29(1)	84(1)		
2002	177(1)		17(1)			191(1)	87(1)		25(1)			
2003			41(3)	196(3)	121(2)	226(1)			27(1)	95(1)		
2004	193(1)	95(1)	49(2)		117(5)	260(1)			31(1)	136(1)		
2005	324(1)	174(1)		243(1)		264(1)	113(1)		33(2)			84(1)
2006	360(2)	139(1)	23(2)		315(2)	345(1)	125(1)		38(1)	98(1)		78(1)
2007	266(2)	205(1)		390(1)	219(4)	319(1)	120(1)		30(1)	136(1)		76(2)
2008	354(2)	392(1)	52(2)		459(5)	396(3)	137(1)	119(1)	30(1)	130(1)		
2009		455(1)		246(1)	110(5)	487(1)	139(1)	29(1)	27(1)		135(1)	76(2)
2010	346(2)	431(2)	49(3)		445(5)	486(1)	136(2)	111(1)	29(1)	158(1)	132(1)	79(2)
2011	342(2)	560(1)	71(3)	639(1)	560(5)	415(1)	195(1)	95(2)	27(1)	138(1)	132(1)	82(2)
2012	285(2)				223(7)	450(1)	297(1)	111(1)	27(1)	109(1)	137(1)	87(2)

### Google Scholar data

For the remaining 17 conferences in the list above, we collected the data using DBLP and Google Scholar. DBLP is a website that lists all papers and authors of a large set of computer science conferences and journals (including all the conferences above). From DBLP, we collected the title of all the papers published in the remaining conference instances from 1996 to 2012. We then selected a random sample of papers listed and queried Google Scholar using the paper’s title. We made sure that we retrieved the citation counts of at least eight papers besides the best paper for each conference instance. The Google Scholar collection was performed in January 2013.

Scholar data refer to 1,577 papers, 170 of which are best papers. Table 2 shows the distribution of the number of papers for each conference instance.

**Table 2:** Specifics of the Google Scholar data. The first figure in each cell is the number of non-best papers in the conference instance. The figure in parenthesis is the number of best papers.

	ACL	CVPR	FOCS	IJCAI	NSDI	OSDI	PLDI	SIGCOMM	SIGIR	SIGMETRICS	SOSP	UIST	VLDB	WWW
1996						15(2)			9(1)	14(2)				
1997				10(3)					8(1)		22(3)		9(1)	
1998									9(1)					
1999				18(2)		9(1)	8(1)		8(1)		29(4)			
2000		9(1)				9(1)	9(1)		8(1)			8(1)		
2001	16(2)			9(1)							17(2)	9(1)	9(1)	
2002			26(3)			9(1)			9(1)			8(1)		9(1)
2003	14(2)		8(1)	18(2)			9(1)		9(1)		23(3)	9(1)		
2004	8(1)		18(2)		9(1)	15(2)	9(1)		9(1)	9(1)		17(2)	9(1)	
2005	8(1)		13(2)	26(3)	9(1)		12(2)		9(1)	9(1)	28(4)	9(1)	9(1)	
2006	9(1)		9(1)		15(2)	16(2)			9(1)			9(1)	9(1)	9(1)
2007	9(1)	9(1)	8(1)	27(3)	9(1)		17(2)		9(1)	9(1)	24(3)	8(1)	8(1)	9(1)
2008	10(2)	18(2)	9(1)		18(2)	25(3)		8(1)	9(1)	8(1)		8(1)		9(1)
2009		9(1)		18(2)	9(1)		9(1)	8(1)	9(1)		27(3)	9(1)		9(1)
2010		9(1)	26(3)		9(1)	18(2)	8(1)	9(1)	9(1)	9(1)		8(1)		9(1)
2011		9(1)	17(2)	27(3)			9(1)	9(1)	9(1)	9(1)	16(2)			
2012			17(2)				9(1)	9(1)	9(1)	9(1)		18(3)		

### Statistical calculations

One of the goals of this research is to evaluate whether a best paper has higher probability of being cited than a random paper from the same conference instance. We call this probability P(best > random). To estimate this probability, we compute for each conference instance the number of pairs of papers (*b*, *nb*) such that *b* is a best paper, *nb* is a non-best paper, and the number of citations of *b* is larger than *nb*, divided by the total number of pairs (*b*, *nb*). Formally, the probability *P*_*c*_ for a conference instance *c* is:

$$\begin{array}{c} P_{c}=\frac{\left|\langle b,nb\rangle \;\text{s.t.}\;cit(b)>cit(nb)\right|}{\left|\langle b,nb\rangle \right|} \end{array}$$

where *cit*(*x*) is the number of citations received by a paper *x*, *b* is a best paper of conference instance *c*, and *nb* is a non-best paper in *c*. If we rank all papers in a conference instance *c* from lowest to highest citation (the higher cited has higher rank), and assign rank = 0 to the lowest cited paper, then the formula for *P*_*c*_ simplifies to:

$$\begin{array}{c} P_{c}=\frac{\sum_{N_{bc}}\text{rank}\left(b_{i}\right)}{N_{bc}*\left(N_{nbc}\right)} \end{array}$$

where *N*_*bc*_ is the number of best papers in *c* and *N*_*nbc*_ is the number of non-best papers in *c*. A similar procedure to estimate probabilities was used in [22].

The final P(best > random) is the average of the proportions for all conference instances (in the same data set). We compute the confidence interval for P(best > random) using a bootstrap procedure on the set of all *P*_*c*_. In particular we used the bias-corrected and accelerated method [23] for calculating confidence intervals and 1000 repetitions for the bootstrap procedure. Intervals reflect a 95% confidence.

To evaluate the evolution of the P(best > random), we perform the procedure separately for the conference instances that took place in the years 2005 to 2011. Since we collected the citations in the beginning of 2013, the calculation of P(best > random) for the year 2011 reflects the citation advantage of best papers two years after they have been selected as best, and the calculation for 2010, reflect the advantage three years after, and so on.

Finally, for how well the best paper selection fared in relation to citations, we used only the Scopus data, and ranked all papers in each of the conferences/year according to its citation count. We computed how many times the best paper was among the top 10% and top 20% cited papers of each conference instance.

We must point out that, in this paper, we do not compare citation counts of different conference instances, for example, we do not compare the citations received by the 2002 AAAI conference with that of the 2010 CIKM or the 2010 AAAI. All comparisons are within a conference instance, thus there is no issue of comparing papers of different years (since the older papers had more chance of receiving citations) or comparing papers in different subareas of Computer Science (different subareas have different citation practices [24] and thus different average citations counts).

The Scopus and Scholar data and programs used for statistical calculations are freely available at Figshare http://dx.doi.org/10.6084/m9.figshare.1147476.

## Results

Using the Scopus data, the probability that a best paper received more citations than a random paper is 0.72 (95% CI = 0.66, 0.77). Using the Google Scholar data that probability is 0.78 (95% CI = 0.74, 0.81). Thus, analysis of both data sets confirm that papers selected as best papers by peer evaluation “are better” than a randomly selected paper from the same conference instance, with 95% confidence.

**Fig 1:**
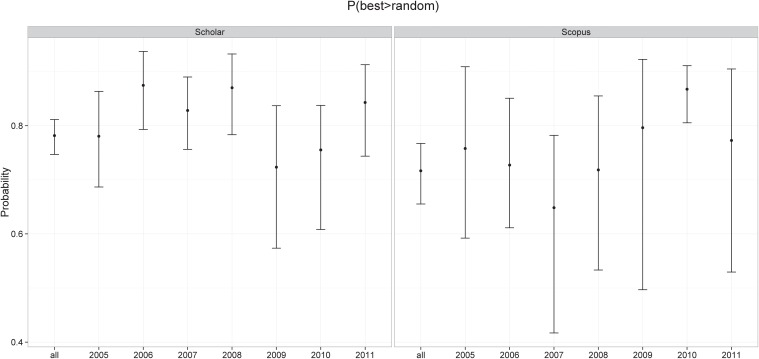
P(best > random) for the two datasets analyzed herein. The entry “all” indicates the overall P(best > random). The error bar indicates the 95% confidence interval and the point at the center indicates the mean value of the probability that a best paper will receive more citations than a random non-best paper. The entries 2005 to 2011 indicate the mean and confidence interval of P(best > random) for conferences that took place in those years.

The temporal evolution of the probability for the years 2005 to 2011 are depicted in Table 3 and in Fig. 1. The figure also displays the overall P(best > random) for both datasets—the first bar in each pane.

**Table 3:** Mean P(best > random) for conferences that took place in the indicated years, for both the Scholar and Scopus datasets.

year	Scholar	Scopus
2005	0.78	0.76
2006	0.87	0.73
2007	0.83	0.65
2008	0.87	0.72
2009	0.72	0.80
2010	0.76	0.87
2011	0.84	0.77

Finally, 51% of the best papers are among the top 10% of the cited papers in each conference instance, while 64% of them are among the top 20%.

## Discussion

Our results show that for Computer Science, the pre-publication exercise of peer evaluation for selecting the best papers is at least better than a random selection, when future citation counts is taken as the gold standard. Or if one takes the dual position, that peer evaluation is the gold standard, then citation count is a reasonable measure of the quality of a paper.

The use of two data sets (and methods) provides independent evidence of the effect. First, the Scopus and the Scholar datasets cover a different set of conferences. Second, Scholar better captures references made by conferences, which as discussed, are an important part of the CS literature. Thus, the most generous explanation to the effect is that the experts that are selecting the best papers do understand the field well and understand the contributions the best paper makes to the field. Notwithstanding there is also a competing explanation that cannot be ruled out at this time, the experts may be selecting highly cited authors with higher probability. Some conferences use a double blind evaluation process for the acceptance/rejection of papers, but it is uncertain whether or not the best paper selection process is blind regarding the authors.

The Scholar data shows a larger P(best > random) than the Scopus data, and we believe there are three alternatives for this difference. The first one is that the conferences in the Scholar data are different than the ones in the Scopus data. Thus it may be the case that the conference set in the Scholar data has a higher P(best > random). The second alternative is that the difference in methodology used in the Scopus and Scholar data is the source of the difference. As discussed above, the Scholar data was sampled, which may introduce bias to the calculation. One such bias is quantization error—since there are much fewer non-best papers in the *P*_*c*_ calculations for the Scholar data, the possible values for *P*_*c*_ are limited to a few values, and that quantization may cause the overall calculation of P(best > random) to be larger. The third alternative is since Scholar collects citations from many more sources than Scopus, these extra citations further compound the advantages of best papers.

To evaluate these three alternatives, we calculated the P(best > random) for the set of conferences instances for which we had both the Scholar and the Scopus data. Notice that these conferences were not in the 17 reported in the Scholar calculations above so that the Scholar data could be considered an independent evaluation of the possible citation advantage of best papers. For a set of 78 conference instances, the Scopus data results in a probability of 0.72 (95%CI = 0.65, 0.77) while the Scholar data results in a probability of 0.77 (95%CI = 0.72, 0.82). The larger result using Scholar data for the same set of conference instances makes it very unlikely that reported overall differences were due to intrinsic differences in the conferences included in each data set.

For the set of 78 conference instances, the median citation count for best papers according to Scopus is 10, and the median citation count for non best papers is 2. For Scholar, the median for best papers is 74.5, and for non-best papers, 17. (We used the median because we are only interested in greater than comparisons, and therefore citations are treated as an ordinal variable for which median is an appropriate centrality measure). The difference between the best and non-best median citations is much larger for Scholar. Although this is not a direct proof that the number of best papers that have more citations than non-best papers is larger in Scholar, it is indirect evidence that points to the conclusion that the higher P(best > random) computed using Scholar citation data is due to the extra citations counted by Scholar. That is, for the sources not indexed by Scopus (other conference and workshop papers, non-indexed journals, theses, technical reports and so on), the citation advantage of best papers is more salient.

An alternative to calculating the advantage of best papers is to compare best papers to papers from the same research group, in the same conference instance, rather than comparing to a random paper from the same conference instance. This was suggested by one of the reviewers of this paper. The rationale, it seems to us, is that if a group received a best paper award, the group is doing quality research in that topic (or in that area of research) and thus the other papers from the same research group should be above average in terms of citations. If this measure, which we will call P(best > same group) is close to 0.5, then what the peers really selected was good research practices or good research groups, and not necessarily “best papers”.

We computed this measure using the Scopus data, with the simple definition that two papers are from the same group if they have at least one author in common. From the 105 conference instances, 52 had best papers that were from the same group as a non-best paper, for a total of 71 best papers and 150 non-best papers. P(best > same group) was calculated as 0.74 (95% CI = 0.64, 0.83). The results are very similar to the overall P(best > random) for the Scopus dataset. Thus it is clear that peers are not selecting either good research practices or good research groups; peers are selecting *papers*, and these papers have statistically higher than 0.5 probability of receiving more citations than both random papers from the same conference, and random papers from the same research group in the same conference.

The temporal evolution of P(best > random) shows that most of the differences between conference instances in a particular year are not statistically different than the overall advantage of best papers. The only result that seems significantly different is for the Scopus dataset for the year 2010. We do not believe there is any real phenomenon for this datapoint; if the citation advantage for best paper is constant across different years (as Fig. 1 indicates) then random variations around the average are expected, and using a confidence value of 95% then, on average, one in 20 of the data points will lay outside the confidence interval. Given that there are 14 datapoints, the apparently odd result for 2010 is not unlikely.

This result indicates that the citation advantage of best papers seems to remain stable at least after two years after the paper was published. Therefore it seems unlikely that the citation advantage of best papers is due to some “propaganda effect”, that is, that the best paper receives more interest and more citations *because* it was selected as a best paper. If the propaganda effect was there and it does not last forever, the advantage of the best paper should have been higher a few years after it was published, and should diminish after the propaganda effect terminates.

There is also some other evidence that there is no propaganda effect for citations of best papers. It is not easy to find out which paper was selected as best for the usual CS bibliographic sources. The official full paper sites for some of the conferences above (ACM portal for the ACM sponsored conferences, and IEEE Explore for the IEEE sponsored conferences) do not list the best papers of the conferences. So an interested researcher would not easily find which were the best papers.

Are best papers really that good? Firstly, best papers are clearly better than random papers from the same conference instance; they have a probability of 0.7 to 0.8 of receiving more citations than a random paper. Secondly, avoiding a naive interpretation that the best paper should receive the most citations, it is clear that papers selected by peers as best papers are probably very good, with over 50% of them being among the top 10% cited papers in the same conference instances.

This research shows evidence that the peer evaluation of best papers in computer science agrees with the results in other fields as reported in [15] and [11].

## References

[1] Oppenheim C (1997) The correlation between citation counts and the 1992 research assessment exercise ratings for British research in genetics, anatomy and archaeology. Journal of Documentation 53: 477–487. 10.1108/EUM0000000007207

[2] Rinia EJ, Van Leeuwen TN, Van Vuren HG, Van Raan AFJ (1998) Comparative analysis of a set of bibliometric indicators and central peer review criteria. Evaluation of condensed matter physics in the Netherlands. Research Policy 27: 95–107. 10.1016/S0048-7333(98)00026-2

[3] Cabezas-Clavijo A, Robinson-García N, Escabias M, Jiménez-Contreras E (2013) Reviewers’ ratings and bibliometric indicators: Hand in hand when assessing over research proposals? PloS one 8: e68258 10.1371/journal.pone.0068258 23840840 PMC3695904

[4] Wainer J, Vieira P (2013) Correlations between bibliometrics and peer evaluation for all disciplines: The evaluation of Brazilian scientists. Scientometrics 96: 395–410. 10.1007/s11192-013-0969-9

[5] Vieira ES, Cabral JA, Gomes JA (2014) How good is a model based on bibliometric indicators in predicting the final decisions made by peers? Journal of Informetrics 8: 390–405. 10.1016/j.joi.2014.01.012

[6] Hirsch JE (2005) An index to quantify an individual’s scientific research output. Proceedings of the National Academy of Sciences of the United States of America 102: 16569–72. 10.1073/pnas.0507655102 16275915 PMC1283832

[7] Bornmann L, Daniel HD (2005) Does the h-index for ranking of scientists really work? Scientometrics 65: 391–392. 10.1007/s11192-005-0281-4

[8] Bornmann L, Mutz R, Daniel HD (2008) Are there better indices for evaluation purposes than the h-index? A comparison of nine different variants of the h-index using data from biomedicine. Journal of the American Society for Information Science and Technology 59: 830–837. 10.1002/asi.20806

[9] Alonso S, Cabrerizo F, Herrera-Viedma E, Herrera F (2009) h-Index: A review focused in its variants, computation and standardization for different scientific fields. Journal of Informetrics 3: 273–289. 10.1016/j.joi.2009.04.001

[10] Sen R, Patel P (2012) Citation rates of award-winning ASCE papers. Journal of Professional Issues in Engineering Education and Practice 138: 107–113. 10.1061/(ASCE)EI.1943-5541.0000092

[11] Coupé T (2013) Peer review versus citations—An analysis of best paper prizes. Research Policy 42: 295–301. 10.1016/j.respol.2012.05.004

[12] Wardle DA (2010) Do ‘faculty of 1000’ (f1000) ratings of ecological publications serve as reasonable predictors of their future impact? Ideas in Ecology and Evolution 3: 11–15.

[13] Bornmann L, Leydesdorff L (2013) The validation of (advanced) bibliometric indicators through peer assessments: A comparative study using data from InCites and F1000. Journal of Informetrics 7: 286–291. 10.1016/j.joi.2012.12.003

[14] Waltman L, Costas R (2014) F1000 recommendations as a new data source for research evaluation: A comparison with citations. Journal of the Association for Information Science and Technology 65: 433–445,. 10.1002/asi.23040

[15] Michayluk D, Zurbruegg R (2014) Do lead articles signal higher quality in the digital age? Evidence from finance journals. Scientometrics 98: 961–973. 10.1007/s11192-013-1115-4

[16] Vardi MY (2009) Conferences vs. journals in computing research. Communications of the ACM 52: 5 10.1145/1592761.1592762

[17] Bar-Ilan J (2010) Web of Science with the conference proceedings citation indexes: The case of computer science. Scientometrics 83: 809–824. 10.1007/s11192-009-0145-4

[18] Franceschet M (2010) The role of conference publications in CS. Communications of the ACM 53: 129 10.1145/1859204.1859234

[19] Halpern JY, Parkes DC (2011) Journals for certification, conferences for rapid dissemination. Communications of the ACM 54: 36 10.1145/1978542.1978555

[20] Freyne J, Coyle L, Smyth B, Cunningham P (2010) Relative status of journal and conference publications in computer science. Communications of the ACM 53: 124–132. 10.1145/1839676.1839701

[21] Wainer J, Goldenstein S, Billa C (2011) Invisible work in standard bibliometric evaluation of computer science. Communications of the ACM 54: 141–146. 10.1145/1941487.1941517

[22] Franceschet M, Costantini A (2011) The first italian research assessment exercise: A bibliometric perspective. Journal of Informetrics 5: 275–291. 10.1016/j.joi.2010.12.002

[23] DiCiccio TJ, Romano JP (1988) A review of bootstrap confidence intervals. Journal of the Royal Statistical Society Series B (Methodological): 338–354.

[24] Wainer J, Eckmann M, Goldenstein S, Rocha A (2013) How productivity and impact differ across computer science subareas. Communications of the ACM 56: 67–73. 10.1145/2492007.2492026

